# ‘S’-shaped curve: modelling trends in smoking prevalence, uptake and cessation in Great Britain from 1973 to 2016

**DOI:** 10.1136/thoraxjnl-2018-212740

**Published:** 2019-08-07

**Authors:** Emma Victoria Beard, Robert West, Martin Jarvis, Susan Michie, Jamie Brown

**Affiliations:** 1 Department of Behavioural Science and Health, University College London, London, UK; 2 Research Department of Clinical, Educational and Health Psychology, University College London, Sheffield, UK

**Keywords:** smoking cessation, tobacco control

## Abstract

**Background:**

It is believed that declines in smoking prevalence naturally slow over time as the smoking population ‘hardens’ and that progress has come primarily from reducing uptake rather than increasing cessation. To address these issues, we undertook the first formal attempt to model the trajectory of smoking prevalence and indices of uptake and cessation in Great Britain from 1973 to 2016.

**Methods:**

Using data from the General Lifestyle Survey between 1973 and 2008, the Integrated Household Survey between 2009 and 2014 and the Annual Population Survey between 2015 and 2016, this study modelled year-on-year changes in smoking prevalence, ever-smoking in 18–24-year-olds as an index of uptake, and quit ratios as an index of cessation.

**Results:**

For all three outcomes, changes over time were best fitted by what may be broadly characterised as ‘S’-shaped curves: segmented functions characterised by initial rapid progress, a slowing or reversal, then renewed progress. Smoking prevalence in Great Britain showed a decelerating decline over time between 1973 and 2000, but then, after the introduction of the National ‘Smoking Kills’ tobacco control plan, the decline accelerated again and has remained nearly linear at −0.67 percentage points per year. Ever-smoking showed a decelerating decline which eventually ceased and began increasing around 1994 but then declined again after 2000. Quit ratios rose rapidly then slowed and then accelerated around 2000 and again more recently in 2013.

**Conclusion:**

Long-term trends in smoking prevalence, uptake and cessation have followed a broadly ‘S’-shaped trend suggesting that they are responsive to major tobacco control initiatives. The decline in prevalence has resulted both from reductions in uptake and increases in cessation.

**Key messages**

**What is the key question?**

- What functions best characterise the trends in smoking prevalence, uptake (indexed by ever-smoking in young adults) and cessation (indexed by quit ratios) from 1973 to 2016 in Great Britain?

**What is the bottom line?**

- Long-term trends in smoking prevalence, uptake and cessation in Great Britain have followed a broadly ‘S’-shaped trend suggesting that they may be responsive to major tobacco control initiatives.

**Why read on?**

- Implementation of comprehensive tobacco control plans appear to bear fruit in Great Britain even as smoking prevalence reaches historic lows by both preventing young people from taking up smoking and increasing quitting.

## Introduction

Cigarette smoking is one of the leading preventable causes of early death and disability worldwide.^1^ The prevalence of cigarette smoking in Great Britain has fallen considerably from its peak in the early 1970s^2^ but the decline has not been linear and there is debate about how far it has resulted from reductions in smoking initiation versus increases in cessation. This paper reports the first formal analysis of the shape of the smoking prevalence curve over time in Great Britain and corresponding changes in smoking initiation and smoking cessation. Such modelling is important for informing future tobacco control policies internationally.

An issue of enduring contention is whether in countries at latter stages of the smoking epidemic, such as the UK and the US, the decline in smoking prevalence naturally slows as the remaining smoking population ‘hardens’.^3^ ‘Hardening’ is the idea that decreases in prevalence result in a greater proportion of remaining smokers having lower motivation to quit and/or greater dependence. This ‘hardening hypothesis’ is relevant to the debate about how far innovation in tobacco control is required to move to the ‘endgame’,^4^ where only a tiny minority of people smoke. If it does become increasingly difficult to reduce smoking prevalence, it may motivate more stringent policies to prevent smoking or possibly a move towards ‘harm reduction’ where smokers who cannot quit have access to reduced harm products that substitute for cigarettes.^5 6^ Although evidence to date provides little support for this hypothesis at the individual level, this paper will be among the first to assess the hardening hypothesis at a population level in Great Britain.^3^

A second contentious issue is whether declines in smoking prevalence have been primarily driven by lower initiation of smoking (that can be indexed by the ever-smoking prevalence in young adults) rather than increases in quitting (that can be indexed by the quit ratio, the proportion of ever-smokers who no longer smoke).^7 8^ If, as some authorities claim, the only successes to date have been in preventing uptake, it calls into question the considerable investment that has been made in promoting and supporting cessation.^5 7 8^

A further contentious issue that has arisen more recently is whether the increase in prevalence of e-cigarette use in countries such as the UK and the US may be renormalising smoking and preventing declines in smoking prevalence that might otherwise have occurred.^9–12^ Conversely, e-cigarette use may be accelerating the decline in smoking prevalence because of smokers switching to these products.^8 13–15^ If e-cigarettes are promoting smoking cessation, this is important in terms of national and international policies regarding regulation of e-cigarettes.

These issues can be addressed by formally modelling the shape of three population parameters over time: smoking prevalence, ever-smoking prevalence in young adults and the quit ratio. Great Britain (England, Wales and Scotland) has been collecting high-quality survey data on these variables since the early 1970s, and graphs plotting trends over time are published annually. What has not been done is to model the shape of the functions relating these variables to time.

The shape of these functions may also reveal the influence of major shifts in tobacco control policy. The first comprehensive tobacco control plan for Great Britain was published in 1998 and represented a sea change in governmental approaches to tobacco control. It set the scene for a range of policies that have been enacted in subsequent years including bans on tobacco marketing, mass media campaigns, a ban on smoking in indoor public areas, introduction of graphic health warnings on packs, increasing the legal age of sale, rises in taxation and the widespread provision of free smoking cessation support.^16^ If there were a change in the trend before and after the late 1990s, this would be suggestive of a population impact of this policy shift.^17^

Thus, we modelled trends in the prevalence of cigarette smoking, ever-smoking in young people and quit ratios using annual data collected between 1973 and 2016 from the General Household Survey (GHS)/General Lifestyle Survey (GLF), the Annual Population Survey (APS) and the Integrated Household Survey (IHS).^18–21^ These surveys cover the population of Great Britain (England, Wales and Scotland). If substantial changes in the trajectory of these indicators have occurred over time, then a non-linear or segmented regression model should provide a better fit to the data than a linear regression model.^22^ The focus here is on cigarette smoking rather than other forms of tobacco use. Although some people who do not smoke cigarettes use tobacco in another form, this is rare in Great Britain.^23^

This study addressed the following question:

What functions best characterise the trends in smoking prevalence, uptake (indexed by ever-smoking in young adults) and cessation (indexed by quit ratios) from 1973 to 2016 in Great Britain?

## Methods

### Design

Individual-level data were aggregated to produce annual population-level estimates from three sources:

#### GLF formerly known as the GHS 1973–2008 (annual n ~18 000)

The GHS/GLF was a multipurpose continuous national survey of people living in private households in Great Britain conducted by the Office for National Statistics (ONS). The survey started in 1971 and was carried out annually, except for breaks in 1997/1998 and 1999/2000. Questions about smoking behaviour have been asked of GHS respondents aged 16 and over since 1973. Between 1974 and 2000, smoking questions were asked in alternate years. Weighting to compensate for non-response was introduced in 2000.^20^

#### IHS 2009–2014 (annual n ~300 000)

The IHS, which reported between 2009 and 2014, was a composite survey of adults aged 18+ combining questions asked on a number of social surveys conducted by the ONS to produce a data set of ‘core’ variables. The largest component was the APS. The IHS used a multistage population weighting procedure which accounts for probability of selection and adjusts for non-response.^18^

#### APS 2015–2016 (annual n ~260 000)

The APS, initiated in 2004, combines results from five different household surveys in Great Britain: the Labour Force Survey (waves 1 and 5); the English Local Labour Force Survey, the Welsh Labour Force Survey and the Scottish Labour Force Survey. Weighting is used to make the combined samples representative of the population of adults aged 16+. Smoking questions are only asked of those aged 18+.^19^

In order to ensure that the data were comparable across the surveys, estimates from the GHS were restricted to those aged 18+.

These surveys are seen as the ‘gold’ standards for prevalence statistics in England. They produce population-level statistics in line with other surveys and use sampling methodologies to ensure representativeness.^24^

### Measures

The smoking outcomes were derived from three questions with yes/no responses: 1. ‘Have you ever smoked a cigarette, a cigar, or a pipe?’; 2. ‘Do you smoke cigarettes at all nowadays?’ and 3. ‘Have you ever smoked cigarettes regularly?’

*Smoking prevalence*: the proportion of respondents answering ‘yes’ to questions 1 and 2.

*Ever-smoking prevalence in young adults:* the proportion of respondents aged 18–24 years answering ‘yes’ to question 1 and also answering ‘yes’ to question 2 or question 3. After 2010, responses to question 3 were unavailable and ever-smoking prevalence was imputed on the following basis: the proportion of respondents answering ‘yes’ to question 1 minus a correction estimate. The correction was the mean difference between ever-smoking prevalence from the GHS and the proportion answering‘yes’ to question 1 from IHS during the 2 years both surveys were conducted (2009 and 2010). Smoking uptake after the age of 24 is extremely rare in the UK so ever-smoking up to that point should capture almost all uptake. While the ever-smoking rate in young adults in a given year does not provide a direct measure of uptake in that year, changes in this figure year on year provide a population-level indication of changes in uptake.

*Quit ratio*: the ratio of ex-regular smoking prevalence (the proportion answering ‘no’’ to question 2 and ‘yes’ to questions 1 and 3) to ever-smoking prevalence. Ex-regular smoking prevalence was also imputed after 2010 to account for the absence of responses to question 3 by the proportion answering ‘no to question 2 and ‘yes’ to question 1 minus a correction estimate. The correction was the mean difference between ex-regular smoking prevalence from the GHS and the proportion answering ‘no’ to question 2 and ‘yes’ from IHS during the 2 years both surveys were conducted (2009 and 2010). Quit ratios do not provide a direct estimate of the quitting rates in a given year but changes in quit ratios provide a population-level index of year-on-year changes in quitting rates.

### Analysis

The analysis plan was preregistered on the Open Science Framework (https://osf.io/8gsk7/). Data were analysed in R V.3.4.0. STrengthening the Reporting of OBservational studies in Epidemiology (STROBE) guidelines were followed throughout.^25^

#### Unsegmented regression

First, time was regressed onto cigarette smoking prevalence, ever-smoking prevalence and quit ratios in a simple linear regression model. Next, several additional models were assessed: (1) polynomial regression with terms up to an order of 3 (ie, quadratic trend and cubic trend model); (2) power regression (log–log model or power trend model); (3) exponential regression (log-level model or exponential trend model) and (4) logarithmic regression (log-level model or logarithmic trend model). Other functions were excluded a priori, for example quartic and quantic polynomial regressions, as they were not believed to reflect plausible underlying trends in prevalence indicators and could lead to overfitting.

The presence of autoregressive-1 autocorrelation [AR(1)] was assessed with the Durbin-Watson test and AR(2) and moving average −1 and −2 [MA(1) and MA(2)] autocorrelation with the autocorrelation function (ACF) and the partial ACF. Higher order AR and MA terms were excluded a priori as they were not believed to be plausible. AR(1) is the most common type of autocorrelation. The Durbin-Watson statistic indicated that AR(1) autocorrelation was present for nearly all of the models that is, observed values at time t influenced observed values in the subsequent time period (t+1) and values observed during that period affected the next period (t+2) and so on (see online supplementary table 1). Where autocorrelation was present, the analysis was repeated using generalised least squares.

10.1136/thoraxjnl-2018-212740.supp1 Supplementary data

#### Segmented regression

The above analyses were then repeated with segmented regression models. These allow relationships that are segmented linear, namely represented by at least two lines connected at ‘breakpoints’. Breakpoints were determined using an iterative procedure, whereby models with different numbers of breaks and positions of breakpoints (up to a maximum of two to prevent overfitting and to be synonymous with the polynomial models) were compared using the Akaike information criterion (AIC). The adjusted R^2^ and Bayesian information criterion (BIC) were used as secondary indices. In general, the smaller the AIC and BIC, and larger the adjusted R^2^, the better the model fit.

#### Model selection

To identify the best overall models, all the resulting regression models were compared using the AIC as the primary measure of fit, and the adjusted R^2^ and BIC as secondary measures of fit (see online supplementary table 2). A prerequisite in using the AIC and BIC to compare models is that the dependent variable is on the same scale; thus, to ensure equivalence for the exponential trend and power trend models, a correction was applied to the AIC and BIC. This involves adding the Jacobian of the log transformation that is, $2\sum_{i}log\left(y_{i}\right)$ where y is the outcome variable of interest. The criteria for selecting the best fitting model was either the model with the lowest AIC, or the simplest model if it was within two units of the model with the lowest AIC score.

Primary interpretation of the results are based on the best fitting model (see table 1). For each outcome, the parameters relating to the linear and both the best fitting unsegmented and segmented models are reported and additionally compared with evidence ratios (ERs, see online supplementary table 4). ERs were calculated as 1/(exp(–(1/2)∆)) with ∆ the difference in AIC. ERs were also provided to compare the best fitting and simple linear models. Model fit indices for all the models are shown in online supplementary table 2 (page 2 of supplementary materials) and shown graphically in online supplementary figures 1-4 (pages 6–9 of supplementary materials). Online supplementary table 3 (page 3 of supplementary materials) provides an overview of the interpretation of the coefficients from the assessed models. Orthogonal polynomials were used for model selection as they are uncorrelated but raw polynomials were reported for the final models.

**Table 1:** Parameters for the best fitting segmented regression models of smoking prevalence, ever-smoking prevalence in young adults and quit ratios in Great Britain between 1973 and 2016

	Smoking prevalence				Ever-smoking prevalence in young adults				Quit ratios			
	Β	95% CI		P value	Β	95% CI		P value	Β	95% CI		P value
Intercept	47.548	45.951	49.144	<0.001	57.667	56.466	58.867	<0.001	25.573	22.698	28.448	<0.001
Time	−1.456	−1.719	−1.193	<0.001	−1.608	−1.840	−1.376	<0.001	1.868	1.399	2.336	<0.001
Time^2^ 0 to BP1	0.026	0.017	0.035	<0.001	0.050	0.041	0.059	<0.001	−0.039	−0.055	−0.023	<0.001
Time^2^ BP1 to BP2	−0.191	−0.345	−0.037	0.017	−0.168	−0.196	−0.139	<0.001	0.059	0.023	0.095	0.002
Time^2^ BP2 to BP3	0.167	0.009	0.342	0.062	0.140	0.103	0.177	<0.001	0.527	0.080	0.975	0.023

Smoking prevalence, quadratic model with two breakpoints in 2000 (year 27) and 2001 (year 28); ever-smoking prevalence in young adults, quadratic model with two breakpoints in 1994 (year 21) and 2002 (year 29); quit ratio, quadratic model with two breakpoints in 1996 (year 23) and 2013 (year 40).

#### Amendments to preregistered analysis plan

In the preregistered analysis, we planned to stratify the analyses by gender, age and socioeconomic status; however, it was clear during the analysis that small sample sizes among some subgroups were creating too much noise for accurate function estimation. Second, data become available for 2016 before the analysis was completed and so this was included to increase relevance. Third, ever-smoking prevalence was originally estimated for all adults but on advice from an expert colleague, we restricted it to 18–24 year olds so that it better reflected recent uptake of tobacco which is usually established by the mid-20s in Great Britain.^26^ Fourth, ex-smoking prevalence mixes ever-smoking and quitting and is reported only in supplementary tables. Finally, we planned to undertake a simple correlation between year and change in smoking prevalence from the previous year. This would give a broad indication as to whether the change had decreased year on year. This analysis was undertaken but given the non-linear nature of the prevalence change (see below), the results are not presented in the main paper (see online supplementary table 5).

#### Confounding by the use of different surveys

Two years of overlapping data collection were available for the IHS and GLF in 2009 and 2010. These suggested that the surveys produced relatively similar prevalence statistics (eg, smoking prevalence IHS *2009 21.5% and 2010 21.1* *%* vs GLF *2009 21.7% and 2010 21.0%*). A sensitivity analysis was run with a dummy variable coded 1 each time data from a new survey was used and 0 all other times. This did not find a significant association between survey use and prevalence of smoking (B=−0.283, 95% CI, −21.812 to 3.246, p=0.141), ever-smoking prevalence in young adults (B=−11.439, 95% CI, −23.645 to 0.767, p=0.065) or quit ratios (B=9.359, 95% CI, –3.535 to 22.252, p=0.148). If the survey changes in survey use had an impact on the measurement of the variables of interest, we would also have expected breakpoints to be identified during the years in which the changes occurred.

### Data sharing

Data are available on the Open Science Framework (https://osf.io/qpxg3/).

### Patient involvement

No patients were involved in setting the research question or the outcome measures, nor were they involved in developing plans for recruitment, design or implementation of the study. No patients were asked to advise on interpretation or writing up of results. There are no plans to disseminate the results of the research directly to study participants or any specific patient community.

## Results

**Figure 1:**
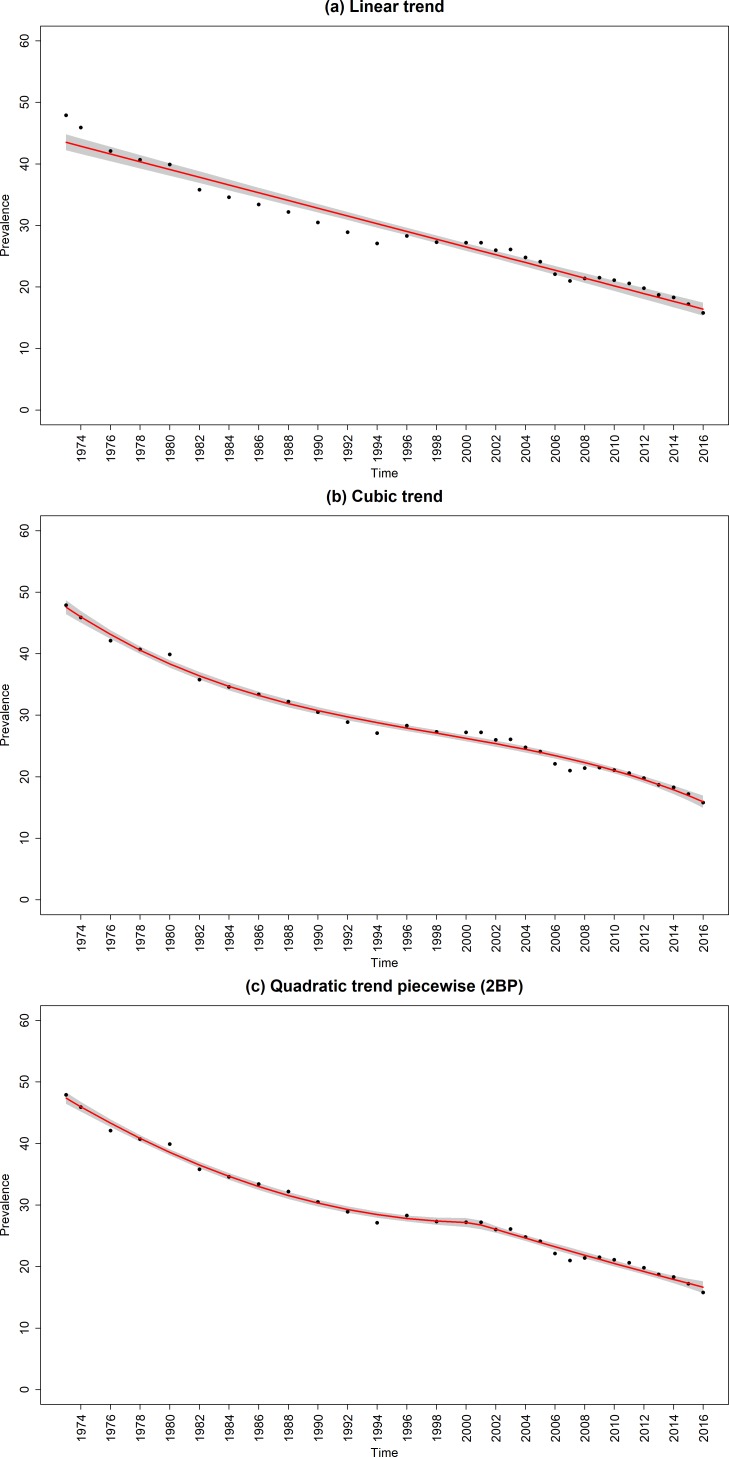
Raw and fitted smoking prevalence from the (a) linear and best fitting (b) non-segmented and (c) segmented regression models for the sample. Red line, regression line; black dots, observed data; shaded grey areas, CIs of regression lines; 2BP, two break points.

**Figure 2:**
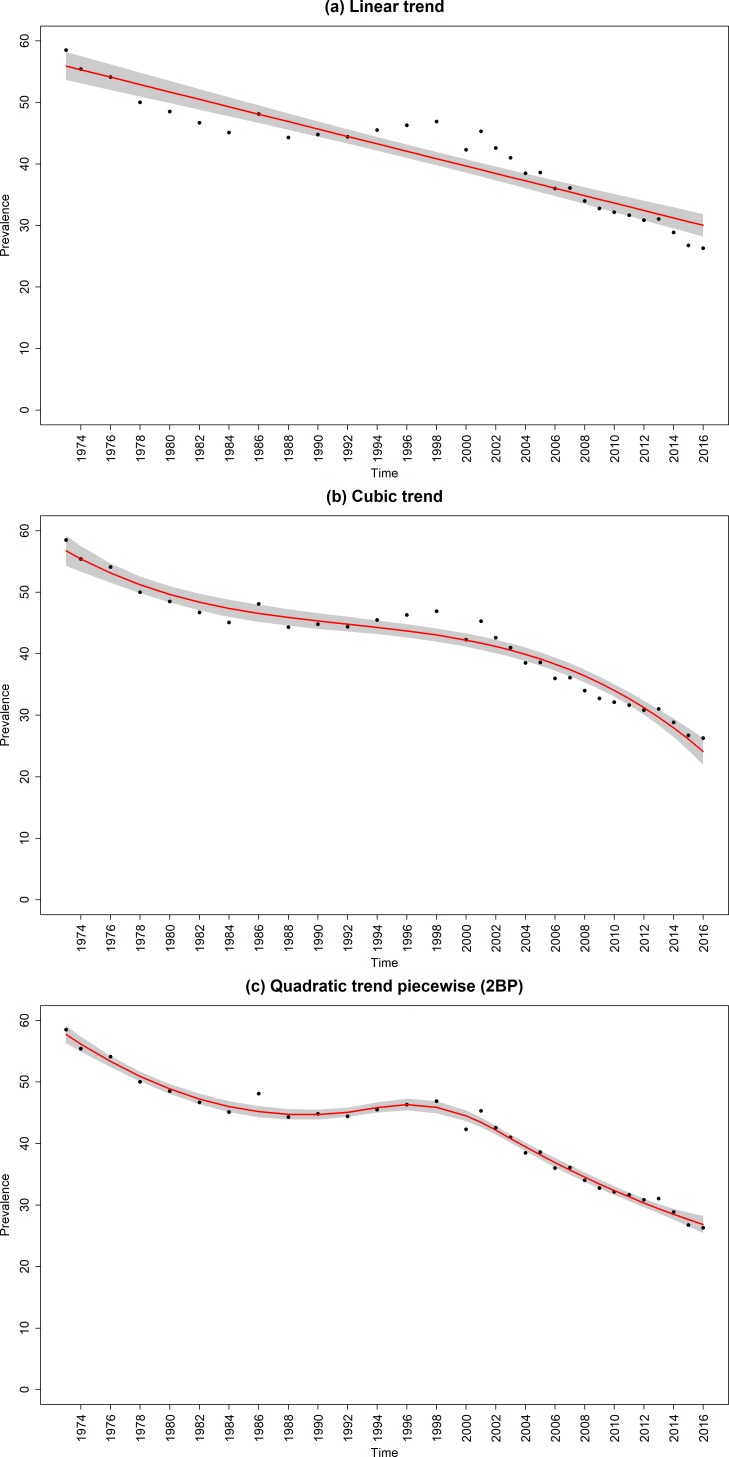
Raw and fitted ever-smoking prevalence in young adults from the (a) linear and best fitting (b) non-segmented and (c) segmented regression models. Red line, regression line; black dots, observed data; shaded grey areas, CIs of regression lines; 2BP, two break points.

**Figure 3:**
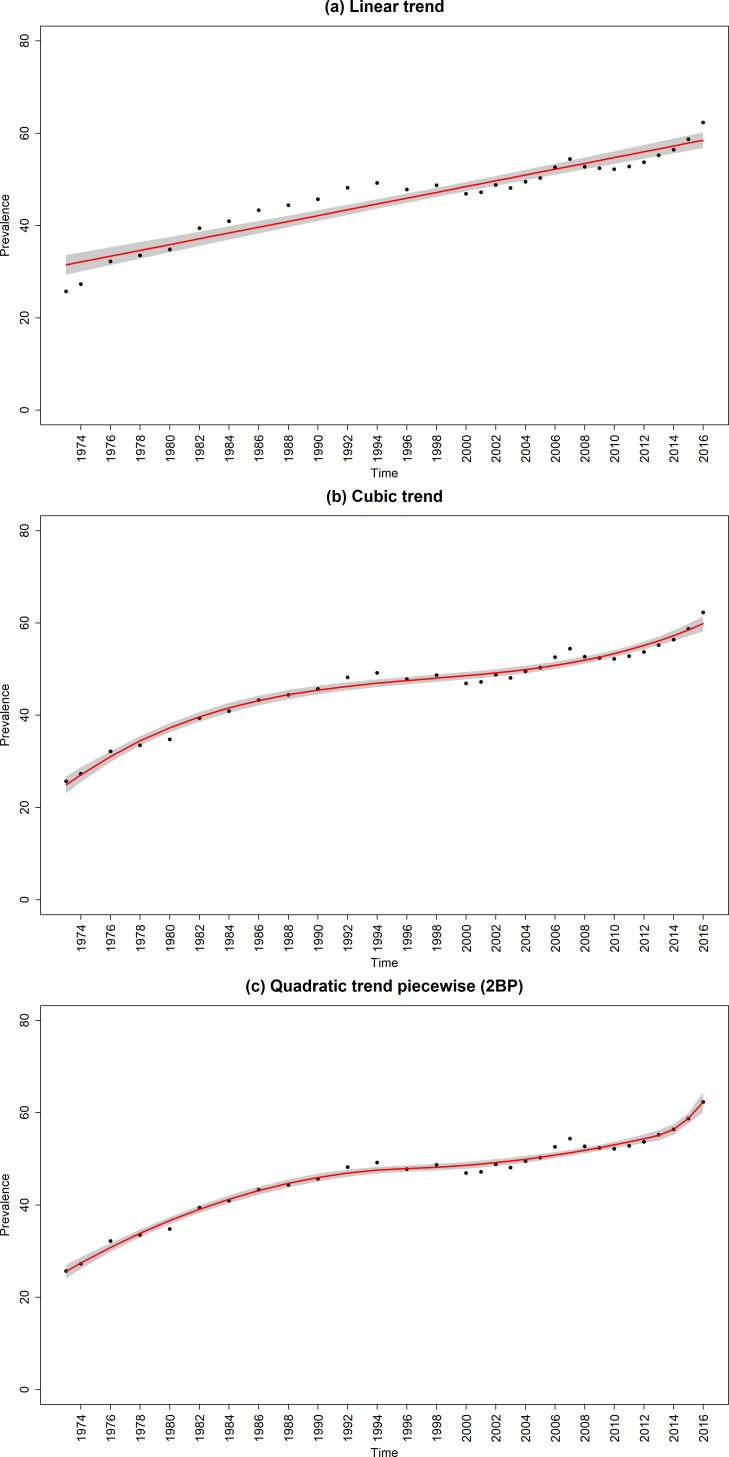
Raw and fitted quit ratios from the (a) linear and best fitting (b) unsegmented and (c) segmented regression models. Red line, regression line; black dots, observed data; shaded grey areas, CIs of regression lines; 2BP, two break points.

The data points in figures 1–3 are the raw time-series data from 1973 to 2016. Prevalence declined from 47.9% (95% CI, 47.3% to 48.5%) in 1973 to 15.8% (95% CI, 15.6% to 16.0%) in 2016; ever-smoking prevalence in young adults decreased from 58.5% (95% CI, 57.9% to 59.1%) to 26.3% (95% CI, 26.1% to 26.5%) and the quit ratio increased from 25.7% (95% CI, 25.1% to 26.3%) to 62.3% (95% CI, 62.1% to 62.5%).

Below is a summary of the results aiming to identify the shape that best characterises the trends in smoking prevalence, uptake (indexed by ever-smoking) and cessation (indexed by quit ratios).

### Smoking prevalence

The best fitting unsegmented regression model was the cubic trend model (figure 1 and online supplementary table 4), while the best fitting model overall was a segmented quadratic trend model with two breakpoints (figure 1 and table 1). This segmented model indicated that from 1973 until to 2000, there was a decelerating decline in smoking prevalence in Great Britain which averaged out at −0.75 percentage points per year over this period with little or no decline at the end. For the next 2 years, there was an accelerating decline in prevalence. After this, prevalence declined in an almost linear manner averaging out at −0.67 percentage point per year from 2001 to 2016.

### Ever-smoking prevalence in young adults

The best fitting unsegmented regression model was the cubic trend model (figure 2 and online supplementary table 4), while the best fitting model overall was a segmented quadratic trend model with two breakpoints (figure 2 and table 1). This segmented model showed a declining quadratic trend in ever-smoking in young adults between 1973 and 1994 so that by the end of the period, it was starting to rise. The rise continued and then reversed around 2000 resulting in a downwardly accelerating quadratic trend to 2002. Since then the decline was nearly linear averaging out at −1.09 percentage points each year.

### Quit ratios

The best fitting standard regression model was the cubic trend model (figure 3 and online supplementary table 4) and best fitting model overall was a segmented quadratic trend model with two breakpoints (figure 3 and table 1). This segmented model showed there was a decelerating increase in quit ratios as part of a quadratic trend between 1973 and 1996, averaging out at 0.93 percentage points per year over the period. This was followed by a slower but accelerating increase from 1996 and 2013 averaging out at 0.42 percentage points over the period. Then, since 2013, there was a sharp rise in quit ratio averaging out at 2.40 percentage points per year.

## Discussion

### Principal findings

Trends in smoking prevalence, uptake and cessation in Great Britain between 1973 and 2016 followed broadly ‘S’-shaped curves. The decline in smoking prevalence in Great Britain followed a decelerating quadratic decline over time from 1973 until 2000. For the following 2-year period, there was an accelerating decline in prevalence. After this, prevalence declined at a nearly linear rate. Ever-smoking in young adults showed a similar pattern of a slowing decline with even evidence for an increase in the latter part of 1990s before showing a nearly linear decline during the first part of the 21st century. Quit ratios showed a decelerating increase and then a period of accelerating increase followed in 2013 by a rapid acceleration.

### Limitations

There are several limitations to the current analysis. First, data were only available annually or once every 2 years and more nuanced trends may have been missed. After 2010, the question ‘Have you ever smoked cigarettes *regularly*?’ was unavailable, which meant that ever-smoking prevalence and quit ratios could not be derived using the same method across the whole time series. However, before its removal, two national surveys ran in parallel—one with and one without the additional question—which allowed an estimate of the difference produced by the two methods and for the figures after 2010 to be inferred. Second, although the finding of a change in trend pre-2000 and post-2000 is indicative of an impact of the change in tobacco control policies, there is a need to assess this directly using time-series analysis in order to draw firmer conclusion.^27^ Third, weighting was only introduced into the methodology in 2000, with unweighted data available for the GHS before this time. However, the effect of this weighting on smoking data has been shown to be small, as weighting reduces the contribution to the overall figure of those aged 60 and over, among whom prevalence is relatively low.^28^ Fourth, we modelled trends in absolute changes of current and ever-smoking prevalence. Linear declines in absolute prevalence figures correspond with increasing relative declines in those measures, which may be obscured by the present analyses. Finally, the identification of breakpoints in segmented models is subject to a degree of uncertainty. However, the fit of all models was very high and the segmented models fit better than the unsegmented models, even taking account of the increased numbers of parameters.

### Conclusion and policy implications

The data clearly conflict with the view that countries at a later stage of the tobacco epidemic necessarily experience slower declines in prevalence as the smoking population ‘hardens’.^3^ Instead, prevalence appears responsive to major tobacco control initiatives. The 1990s saw a period of stagnation in decline in smoking prevalence and smoking uptake even began to rise. But from around 2000 onwards, when the sea change in tobacco control activity occurred in Great Britain, progress in reducing smoking prevalence was reinitiated.^16 29 30^ The acceleration in quit ratios and declines in ever smokers during this period broadly mirrored the trends in prevalence reduction.^31^

The persistence of a near linear decline in smoking prevalence does not support concerns that increase in prevalence of e-cigarette use—as has been observed in Great Britain since 2011—would renormalise smoking and prevent declines that might otherwise have occurred.^9–12^ In fact, the trend observed is consistent with a time series analysis conducted in 2016 over a time period of 10 years, which suggested that e-cigarettes have contributed to the decline in smoking prevalence by helping some smokers to quit successfully.^13^ That study showed an increase in the success rate of quit attempts associated with the increase in prevalence of e-cigarette use, after adjusting for a wide range of potentially confounding factors including policy initiatives.^13^

The current results show that a reduction in uptake and an increase in cessation have both contributed to the decline of smoking prevalence in Great Britain, and conflicts with the view that declines have been largely driven by reductions in uptake of smoking rather than increases in quitting.^7 8^ In this respect, Great Britain appears similar to the USA, which also achieved substantial declines in prevalence between 1990 and 2014 by improvements in quitting.^32^ Analyses indicating that there has been little or no progress in quitting in Great Britain have focused on quit rates as a proportion of the *adult population* rather than as a proportion of *ever-smokers*.^7^ This fails to take account of the fact that someone who has never smoked cannot become an ex-smoker.

In conclusion, long-term trends in smoking prevalence in Great Britain broadly follow an ‘S’-shaped curve. They do not support the view that the decline necessarily slows or that smokers necessarily become resistant quitting, but rather suggests that it is responsive to major tobacco control initiative. The prevalence decline resulted from both a reduction in uptake and an increase in cessation.

## References

[1] Peacock A, Leung J, Larney S, et al Global statistics on alcohol, tobacco and illicit drug use: 2017 status report. Addiction 2018;113:1905–26. 10.1111/add.14234 29749059

[2] Brown J, West R Smoking prevalence in England is below 20% for the first time in 80 years. BMJ 2014;348 10.1136/bmj.g1378 24519763

[3] Hughes JR The hardening hypothesis: is the ability to quit decreasing due to increasing nicotine dependence? A review and commentary. Drug Alcohol Depend 2011;117:111–7. 10.1016/j.drugalcdep.2011.02.009 21411244 PMC3133840

[4] Chapman S, Wakefield MA Large-scale unassisted smoking cessation over 50 years: lessons from history for endgame planning in tobacco control. Tobacco Control 2013;22(suppl 1):i33–5. 10.1136/tobaccocontrol-2012-050767 23591504 PMC3632984

[5] Tobacco Advisory Group of the Royal College of Physicians Hiding in plain sight treating tobacco dependency in the NHS, 2018.

[6] Department of Health and Social Care Towards a smoke-free generation: tobacco control plan for England, 2017 Available: https://www.gov.uk/government/publications/towards-a-smoke-free-generation-tobacco-control-plan-for-england

[7] Nicotine without smoke-tobacco harm reduction Royal College of physicians, 2016.

[8] Britton J, Arnott D, McNeill A, et al Nicotine without smoke-putting electronic cigarettes in context. BMJ 2016;353 10.1136/bmj.i1745 27122374

[9] Kalkhoran S, Glantz SA E-cigarettes and smoking cessation in real-world and clinical settings: a systematic review and meta-analysis. Lancet Respir Med 2016;4:116–28. 10.1016/S2213-2600(15)00521-4 26776875 PMC4752870

[10] Chapman S E-cigarettes: the best and the worst case scenarios for public health--an essay by Simon Chapman. BMJ 2014;349 10.1136/bmj.g5512 25204397

[11] Cataldo JK, Petersen AB, Hunter M, et al E-cigarette marketing and older smokers: road to renormalization. Am J Health Behav 2015;39:361–71. 10.5993/AJHB.39.3.9 25741681 PMC4351761

[12] Kalkhoran S, Glantz SA Modeling the health effects of expanding e-cigarette sales in the United States and United Kingdom: a Monte Carlo analysis. JAMA Intern Med 2015;175:1671–80. 10.1001/jamainternmed.2015.4209 26322924 PMC4594196

[13] Kuipers MAG, Beard E, Hitchman SC, et al Impact on smoking of England's 2012 partial tobacco point of sale display ban: a repeated cross-sectional national study. Tob Control 2017;26 10.1136/tobaccocontrol-2015-052724 PMC6166602 26903596

[14] Levy DT, Borland R, Villanti AC, et al The application of a Decision-Theoretic model to estimate the public health impact of Vaporized nicotine product initiation in the United States. NICTOB 2017;19:149–59. 10.1093/ntr/ntw158 PMC5234365 27613952

[15] Cherng ST, Tam J, Christine PJ, et al Modeling the effects of e-cigarettes on smoking behavior: implications for future adult smoking prevalence. Epidemiology 2016;27:819–26. 10.1097/EDE.0000000000000497 27093020 PMC5039081

[16] Action on smoking and health Smoking still kills, 2015 Available: http://www.ncsct.co.uk/usr/pub/Smoking%20Still%20Kills.pdf

[17] Beard E, Brown J, McNeill A, et al Has growth in electronic cigarette use by smokers been responsible for the decline in use of licensed nicotine products? Findings from repeated cross-sectional surveys. Thorax 2015;70:974–8. 10.1136/thoraxjnl-2015-206801 26209508 PMC4602271

[18] Office for National Statistics The National archives: integrated Household Survey, 2016 Available: http://www.ons.gov.uk/ons/guide-method/method-quality/specific/social-and-welfare-methodology/integrated-household-survey/index.html

[19] Office for national statistics Annual population survey, 2012 Available: https://www.ons.gov.uk/employmentandlabourmarket/peopleinwork/employmentandemployeetypes/qmis/annualpopulationsurveyapsqmi

[20] Office for national statistics. opinions and lifestyle survey. Secondary Opinions and Lifestyle Survey 2016 https://www.ons.gov.uk/surveys/informationforhouseholdsandindividuals/householdandindividualsurveys/opinionsandlifestylesurveyopn

[21] Office for National Statistics The National Archieves: general lifestyle survey. General Lifestyle Survey: Secondary The National Archieves, 2016.

[22] Muggeo VM Segmented: an R package to fit regression models with broken-line relationships. R news 2008;8:20–5.

[23] Office for National Statistics Smoking and drinking among adults, 2009. Secondary smoking and drinking among adults, 2009 2009. Available: https://www.ons.gov.uk/ons/./2009./smoking-and-drinking-among-adults--2009.pdf

[24] Fidler JA, Shahab L, West O, et al 'The smoking toolkit study': a national study of smoking and smoking cessation in England. BMC Public Health 2011;11 10.1186/1471-2458-11-479 PMC3145589 21682915

[25] von Elm E, Altman DG, Egger M, et al The strengthening the reporting of observational studies in epidemiology (STROBE) statement: guidelines for reporting observational studies. Int J Surg 2014;12:1495–9. 10.1016/j.ijsu.2014.07.013 25046131

[26] Hopkinson NS, Lester-George A, Ormiston-Smith N, et al Child uptake of smoking by area across the UK. Thorax 2014;69:873–5. 10.1136/thoraxjnl-2013-204379 24304854 PMC4145434

[27] Langley T, Szatkowski L, Lewis S, et al The freeze on mass media campaigns in England: a natural experiment of the impact of tobacco control campaigns on quitting behaviour. Addiction 2014;109:995–1002. 10.1111/add.12448 24325617

[28] Office for National Statistics Smoking and drinking among adults, 2007, 2017 Available: https://www.le.ac.uk/users/dsgp1/COURSES/THIRDMET/EXERCISES/SmokeDATA.pdf

[29] West R, May S, West M, et al Performance of English stop smoking services in first 10 years: analysis of service monitoring data. BMJ 2013;347 10.1136/bmj.f4921 23963106

[30] Thomas S, Fayter D, Misso K, et al Population tobacco control interventions and their effects on social inequalities in smoking: systematic review. Tobacco Control 2008;17:230–7. 10.1136/tc.2007.023911 18426867 PMC2565568

[31] ASH Key dates in the history of anti-tobacco campaigning, 2017 Available: http://ash.org.uk/information-and-resources/briefings/key-dates-in-the-history-of-anti-tobacco-campaigning/

[32] Mendez D, Tam J, Giovino GA, et al Has Smoking Cessation Increased? An Examination of the US Adult Smoking Cessation Rate 1990 - 2014. Nicotine Tob Res 2016. 10.1093/ntr/ntw239 27634956

