# Supplementary material for: ‘S’-shaped curve: modelling trends in smoking prevalence, uptake and cessation in Great Britain from 1973 to 2016
Source: Thorax. 2019 Aug 7;74(9):875–81. doi: 10.1136/thoraxjnl-2018-212740 (PMC6824614; doi:10.1136/thoraxjnl-2018-212740)

Supplementary Table 1: Results of the autoregression analysis

**Smoking prevalence**			**Ever smoking prevalence (18-24 year olds)**			**Regular ex-smoking prevalence**			**Quit ratios**		
DW	*P*	Autocorrelation	DW	*p*	Autocorrelation	DW	*p*	Autocorrelation	DW	*p*	Autocorrelation
0.393	<0.001	AR(1)	0.413	<0.001	AR(1)	0.205	<0.001	AR(1)	0.334	<0.001	AR(1)

Note: DW=Durbin-Watson statistics; PACF=partial autocorrelation function; ACF=autocorrelation function; AR=autoregressive autocorrelation; MA=moving average autocorrelation; DW only tests for lag-1 AR autocorrelation; the PACF and ACF were used to assess MA(1), MA(2) and AR(2).

Beard EV, *et al. Thorax* 2019; 0:875–881. doi: 10.1136/thoraxjnl-2018-212740

Supplementary Table 2: Indices of fit for the regression models (not accounting for autocorrelation)

Model	Smoking prevalence			Ever smoking prevalence (18-24 years old)			Regular ex-smoking prevalence			Quit ratios		
	AIC	BIC	Adj. R^2	AIC	BIC	Adj. R^2	AIC	BIC	Adj. R^2	AIC	BIC	Adj. R^2
Linear trend model	123.2	127.5	0.96	158.4	162.7	0.88	138.1	142.4	0.23	154.2	158.5	0.90
Quadratic trend model	113.7	119.4	0.97	151.9	157.6	0.91	108.4	114.2	0.71	147.8	153.6	0.92
Cubic trend model	83.2	90.3	0.99	131.8	139.0	0.95	73.3	80.5	0.91	114.6	121.7	0.97
Logarithmic trend model	150.4	154.7	0.91	186.1	190.4	0.71	119.8	124.1	0.57	153.0	157.3	0.91
Exponential trend model	111.5	115.8	0.97	167.3	171.6	0.85	142.5	146.8	0.26	172.8	177.1	0.86
Power trend model	168.4	172.7	0.81	195.4	199.8	0.62	121.7	126.0	0.62	136.5	140.8	0.96
Linear piecewise trend model (1BP)	94.6	101.7	0.99	139.1	146.3	0.94	81.4	88.6	0.88	133.2	140.4	0.95
Linear piecewise trend model (2BP)	86.0	96.1	0.99	104.9	114.9	0.98	60.0	70.1	0.94	112.7	122.8	0.98
Quadratic piecewise trend model (1BP)	79.0	86.1	0.99	117.4	124.6	0.97	67.9	75.1	0.92	109.6	116.7	0.98
Quadratic piecewise trend model (2BP)	74.8	83.4	0.99	98.8	107.4	0.98	51.6	60.2	0.96	103.0	111.6	0.98
Cubic piecewise trend model (1BP)	83.9	92.5	0.99	106.4	115.0	0.98	57.9	66.5	0.95	109.1	117.7	0.98
Cubic piecewise trend model (2BP)	80.8	90.8	0.99	102.0	112.0	0.98	56.4	66.4	0.95	108.7	118.7	0.98
Logarithmic piecewise trend model (1BP)	109.2	116.4	0.98	108.6	115.7	0.98	86.0	93.2	0.87	142.5	149.7	0.94
Logarithmic piecewise trend model (2BP)	79.5	89.5	0.99	101.4	111.4	0.98	81.8	91.9	0.89	117.9	127.9	0.97
Exponential piecewise trend model (1BP)	100.0	107.2	0.98	12.07	134.2	0.96	82.0	89.2	0.9	127.3	134.4	0.97
Exponential piecewise trend model (2BP)	80.9	90.9	0.99	101.4	111.5	0.98	64.7	74.7	0.95	116.4	126.4	0.98
Power trend piecewise model (1BP)	119.0	101.7	0.97	139.1	111.1	0.98	81.7	88.9	0.9	122.6	129.7	0.97
Power trend piecewise model (2BP)	95.6	96.1	0.99	104.9	120.7	0.99	64.8	74.9	0.95	126.9	136.9	0.97

Note: Blue indicates the selected model out of all possible models

Beard EV, *et al. Thorax* 2019; 0:875–881. doi: 10.1136/thoraxjnl-2018-212740

Supplementary Table 3: Interpretation of the coefficients from the assessed models

Model	Coefficient	Interpretation
Linear trend model	Intercept	Value of the dependent variable at the start of the series
	Time	Linear slope between time and the dependent variable. If the sign is positive then the dependent variable increases as time increases, if the sign is negative then the dependent variable decreases as time increases.
Quadratic trend model	Intercept	Value of the dependent variable at the start of the series
	Time	Rate of change in smoking prevalence at the start of the series
	$Time^2$	The quadratic trend over the series. If the sign is positive then the model is convex (curvature is upwards) and if it is negative then the curve is concave (curvature is downwards).
Cubic trend model	Intercept	Value of the dependent variable at the start of the series
	Time	Rate of change in smoking prevalence at the start of the series
	$Time^2$	The quadratic trend over the series. If the sign is positive then the model is convex (curvature is upwards) and if it is negative then the curve is concave (curvature is downwards).
	$Time^3$	The cubic trend over the time series. If negative then the quadratic trend is increasingly negative as time increases. If positive then the quadratic trend is increasingly positive as time increases.
Linear piecewise trend model (1 BP)	Intercept	Value of the dependent variable at the start of the series
	Time 0 to BP1	Linear slope between time and the dependent variable from baseline to BP1. If the sign is positive then the dependent variable increases as time increases, if the sign is negative then the dependent variable decreases as time increases
	Time BP2 to end	Change in the linear slope between time and the dependent from BP1 to the end of the series. The linear term for this period is thus Time 0 to BP1 + Time BP2 to end
Quadratic piecewise trend model (1 BP)	Intercept	Value of the dependent variable at the start of the series
	Time	Rate of change in smoking prevalence at the start of the series
	$Time^2$ 0 to BP1	The quadratic trend between 0 and BP1. If the sign is positive then the model is convex (curvature is upwards) and if it is negative then the curve is concave (curvature is downwards).
	$Time^2$ BP1 to end	The change in the quadratic trend between BP1 and the end of the series. The quadratic term for this period is thus $Time^2$ 0 to BP1+ $Time^2$ BP1 to end. The linear term for this period is thus Time 0 to BP1 + Time BP2 to end
Cubic piecewise trend model (1 BP)	Intercept	Value of the dependent variable at the start of the series
	Time	Rate of change in smoking prevalence at the start of the series
	$Time^2$	The quadratic trend over the series. If the sign is positive then the model is convex (curvature is upwards) and if it is negative then the curve is concave (curvature is downwards).
	$Time^3$ 0 to BP1	The cubic trend between baseline and BP1. If negative then the quadratic trend is increasingly negative as time increases. The quadratic trend at smaller values of time is positive (linear slope gets more positive), while the quadratic trend at larger values of time is negative (linear slope gets more less positive). If positive then the quadratic trend is increasingly positive as time increases.
	$Time^3$ BP1 to end	Change in the cubic trend between BP1 and the end of the series. The cubic term for this period is thus $Time^3$ 0 to BP1 + $Time^3$ BP1 to end.

Beard EV, *et al. Thorax* 2019; 0:875–881. doi: 10.1136/thoraxjnl-2018-212740

Supplementary Table 4: Results of the linear model and best fitting standard regression models and piecewise regression models for the sample overall

	Smoking prevalence				Ever smoking prevalence (18-24 year olds)				Regular ex-smoking prevalence				Quit ratios			
	β	95%CI Lower	95%CI Upper	P	β	95%CI Lower	95%CI Upper	P	β	95%CI Lower	95%CI Upper	P	B	95%CI Lower	95%CI Upper	P
**Linear model**																
No autocorrelation																
Intercept (B_0)	43.520	42.225	44.816	<0.001	55.906	53.520	58.192	<0.001	21.411	19.764	23.058	<0.001	31.460	29.323	33.597	<0.001
Time (B_1)	-0.630	-0.677	-0.584	<0.001	-0.602	-0.684	-0.520	<0.001	0.092	0.033	0.151	0.003	0.628	0.552	0.705	<0.001
Autocorrelation																
Intercept (B_0)	47.479	32.902	62.057	<0.001	57.520	47.447	67.592	<0.001	16.353	-8.601	41.307	0.191	26.474	4.818	48.129	0.018
Time (B_1)	-0.737	-0.973	-0.501	<0.001	-0.683	-0.989	-0.377	<0.001	0.236	0.011	0.460	0.040	0.818	0.461	1.174	<0.001
**Best fitting standard model**																
No autocorrelation																
Intercept (B_0)	47.559	46.439	48.679	<0.001	56.729	54.275	59.183	<0.001	15.979	15.023	16.934	<0.001	24.902	23.044	26.760	<0.001
Time (B_1)	-1.619	-1.849	-1.389	<0.001	-1.375	-1.880	-0.871	<0.001	1.265	1.068	1.461	<0.001	2.293	1.911	2.675	<0.001
Time^2 (B_2^2)	0.048	0.036	0.060	<0.001	0.059	0.032	0.086	<0.001	-0.051	-0.061	-0.040	<0.001	-0.083	-0.103	-0.062	<0.001
Time^3 (B_3^3)	-0.001	-0.001	<0.001	<0.001	-0.001	-0.001	-0.001	<0.001	0.001	<0.001	0.001	<0.001	0.001	0.001	0.001	<0.001
Autocorrelation																
Intercept (B_0)	47.754	45.754	49.755	<0.001	57.924	51.709	64.138	<0.001	16.490	11.748	21.232	<0.001	25.597	18.453	32.742	<0.001
Time (B_1)	-1.668	-2.075	-1.260	<0.001	-1.561	-2.657	-0.464	0.007	1.277	0.761	1.792	<0.001	2.318	1.412	3.229	<0.001
Time^2 (B_2^2)	0.050	0.028	0.073	<0.001	0.065	0.003	0.127	0.041	-0.056	-0.085	-0.027	0.001	-0.090	-0.142	-0.039	0.001
Time^3 (B_3^3)	-0.001	-0.001	<0.001	<0.001	-0.001	-0.002	<0.001	0.028	0.001	<0.001	0.001	0.003	0.001	0.001	0.002	0.002
**Best fitting piecewise model**																
No autocorrelation																
Intercept (B_0)	47.384	46.458	48.310	<0.001	57.722	56.295	59.149	<0.001	16.450	15.811	17.088	<0.001	25.613	24.153	27.074	<0.001
Time (B_1)	-1.439	-1.594	-1.284	<0.001	-1.616	-1.894	-1.339	<0.001	0.990	0.884	1.096	<0.001	1.838	1.596	2.079	<0.001
Time^2 0 to BP1 (B_2^2)	0.026	0.020	0.031	<0.001	0.050	0.039	0.061	<0.001	-0.025	-0.029	-0.022	<0.001	-0.038	-0.046	-0.030	<0.001
Time^2 BP1 to BP2 (B_3^2)	-0.359	-0.534	-0.184	<0.001	-0.168	-0.203	-0.133	<0.001	0.026	0.018	0.034	<0.001	0.057	0.039	0.074	<0.001
Time^2 BP2 to BP3 (B_4^2)	0.337	0.151	0.523	0.001	0.140	0.096	0.185	<0.001	0.227	0.138	0.316	<0.001	0.532	0.186	0.879	0.004
Autocorrelation																
Intercept (B_0)	47.548	45.951	49.144	<0.001	57.667	56.466	58.867	<0.001	16.462	15.637	17.287	<0.001	25.573	22.698	28.448	<0.001
Time (B_1)	-1.456	-1.719	-1.193	<0.001	1.608	-1.840	-1.376	<0.001	0.989	0.851	1.128	<0.001	1.868	1.399	2.336	<0.001
Time^2 0 to BP1 (B_2^2)	0.026	0.017	0.035	<0.001	0.050	0.041	0.059	<0.001	-0.025	-0.030	-0.021	<0.001	-0.039	-0.055	-0.023	<0.001
Time^2 BP1 to BP2 (B_3^2)	-0.191	-0.345	-0.037	0.017	-0.168	-0.196	-0.139	<0.001	0.025	0.015	0.036	<0.001	0.059	0.023	0.095	0.002
Time^2 BP2 to BP3 (B_4^2)	0.167	0.009	0.342	0.062	0.140	0.103	0.177	<0.001	0.231	0.124	0.338	<0.001	0.527	0.080	0.975	0.023

Note: *Smoking prevalence: best fitting standard regression model = cubic model (with point of inflection identified by second order derivatives in 1989 (year 16)), best fitting piecewise model = quadratic model with two breakpoints in 2000 (year 27) and 2001 (year 28); Ever smoking prevalence: best fitting standard regression model = cubic model (with point of inflection identified by second order derivatives in 1993 (year 20)), best fitting piecewise model = quadratic model with two breakpoints in 1994 (year 21) and 2002 (year 29); Ex-smoking prevalence: best fitting standard regression model = cubic model (with point of inflection identified by second order derivatives in 1990 (year 17)), best fitting piecewise model = quadratic model with two breakpoints in 1996 (year 23) and 2012 (year 39); Quit ratio: best fitting standard regression model = cubic model (with point of inflection identified by second order derivatives in 2001 (year 28)), best fitting piecewise model = quadratic model with two breakpoints in 1996 (year 23) and 2013 (year 40); NA = not applicable; Evidence ratios (EV): smoking - best fitting standard model versus linear model ER=3.286e+10, best fitting piecewise model versus linear model ER=496208161; Ever smoking - best fitting standard model versus linear model ER=596805, best fitting piecewise model versus linear model ER=8.702e+12; Ex smoking - best fitting standard model versus linear model ER=6.688e+10, best fitting piecewise model versus linear model ER=3.559e+15; Quit Ratio - best fitting standard model versus linear model ER=410095133, best fitting piecewise model versus linear model ER=1.323e+11*

Beard EV, *et al. Thorax* 2019; 0:875–881. doi: 10.1136/thoraxjnl-2018-212740

Supplementary Table 5: Correlation between year and change in smoking prevalence from the previous year

Year	Smoking prevalence difference $t_n - t_{n+1}$
1973	2
1974	3.8
1976	1.4
1978	0.8
1980	4.1
1982	1.2
1984	1.2
1986	1.2
1988	1.7
1990	1.6
1992	1.8
1994	-1.2
1996	1
1998	0.1
2000	0
2001	1.2
2002	-0.1
2003	1.3
2004	0.7
2005	2
2006	1.1
2007	-0.4
2008	-0.1
2009	0.4
2010	0.5
2011	0.8
2012	1.1
2013	0.4
2014	1.1
2015	1.4

There was a significant negative rank order correlation (r=-0.50; 95%CI -0.73 to -0.17, p=0.005) between the annual change in smoking prevalence (i.e. $t_n - t_{n+1}$) and year, which suggests that the decline in overall prevalence has changed significantly over time.

Beard EV, *et al. Thorax* 2019; 0:875–881. doi: 10.1136/thoraxjnl-2018-212740

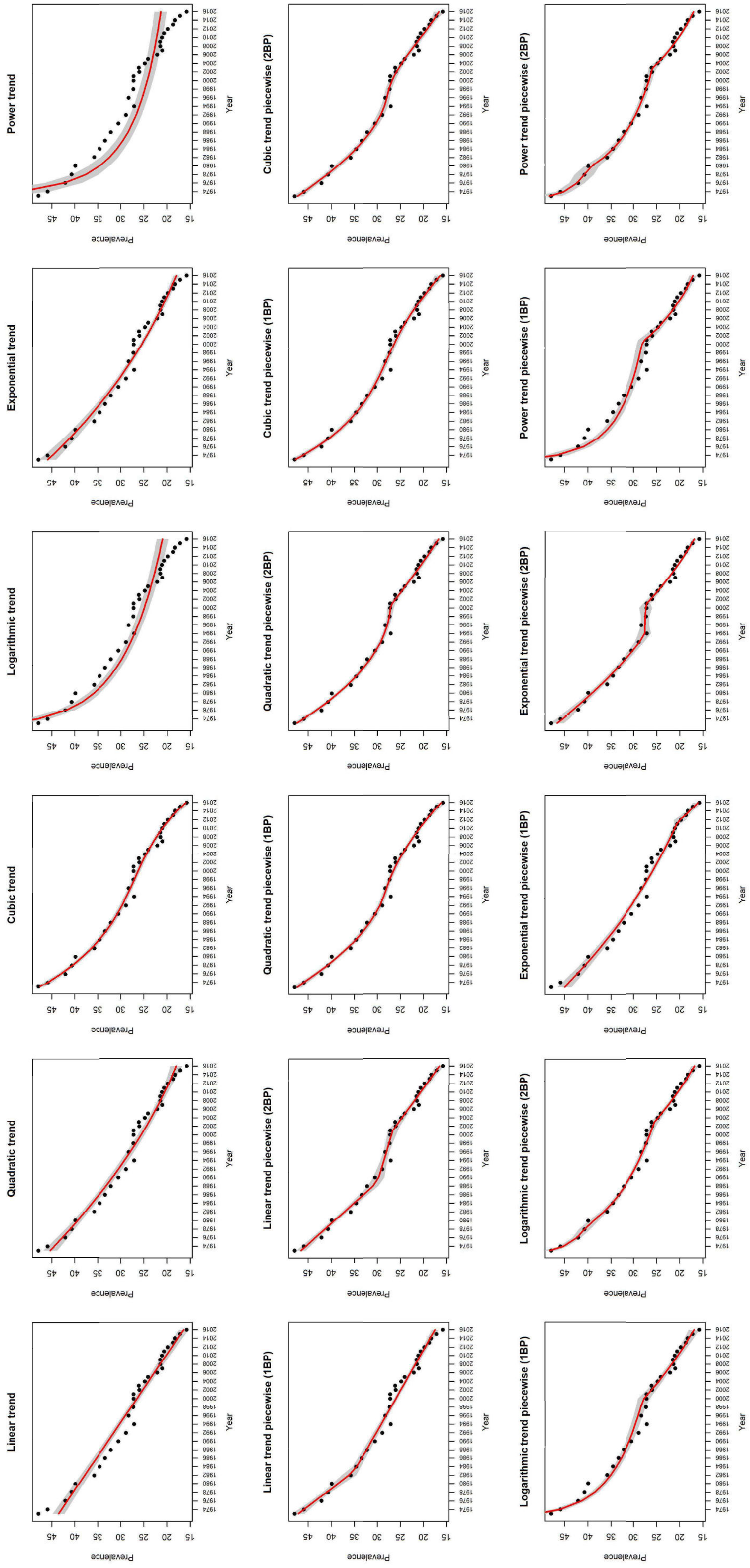

Supplementary Figure 1: Raw and fitted smoking prevalence from the assessed regression models

Supplementary Figure 2: Raw and fitted ever smoking prevalence from the assessed regression models

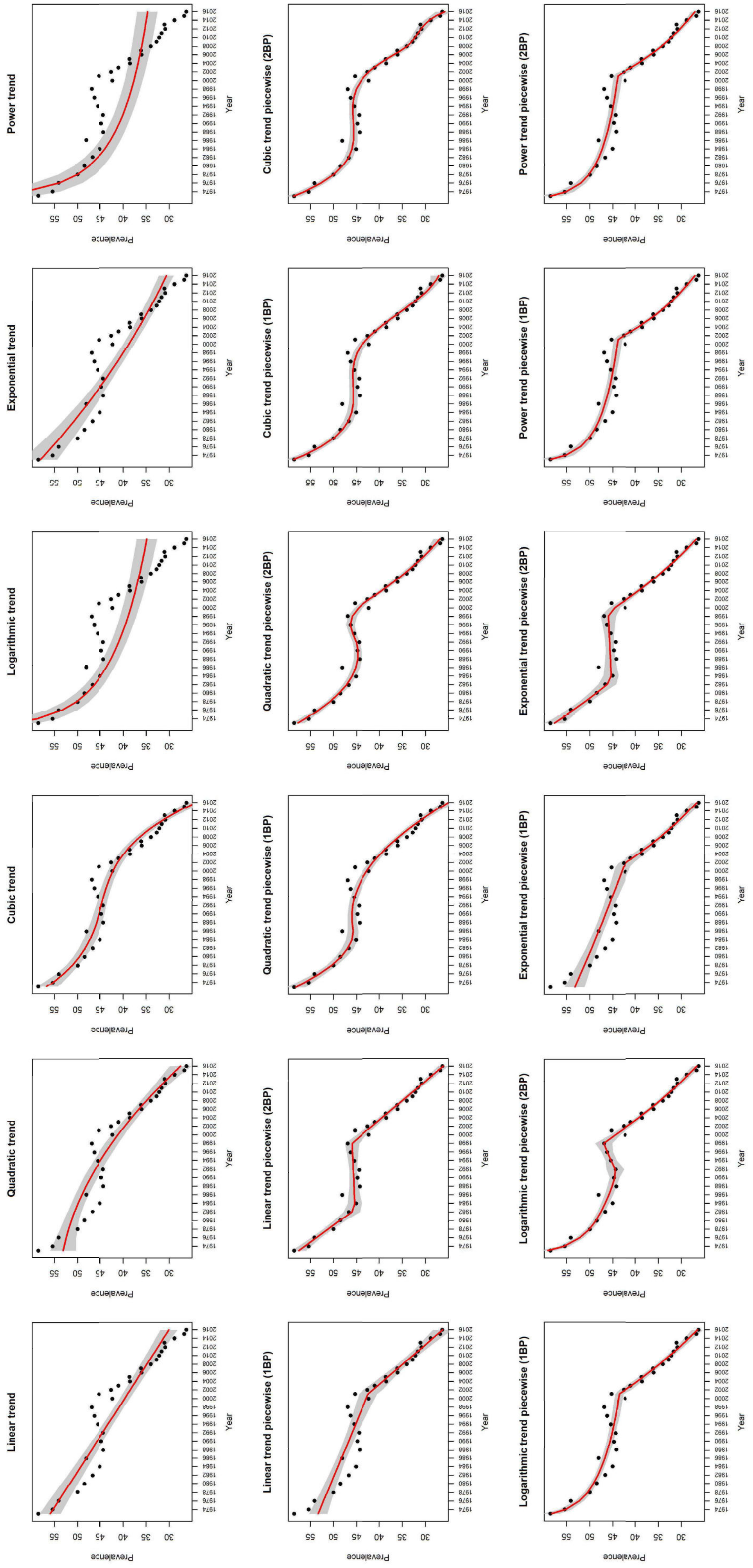

Supplementary Figure 3: Raw and fitted ex-smoking prevalence from the assessed regression models

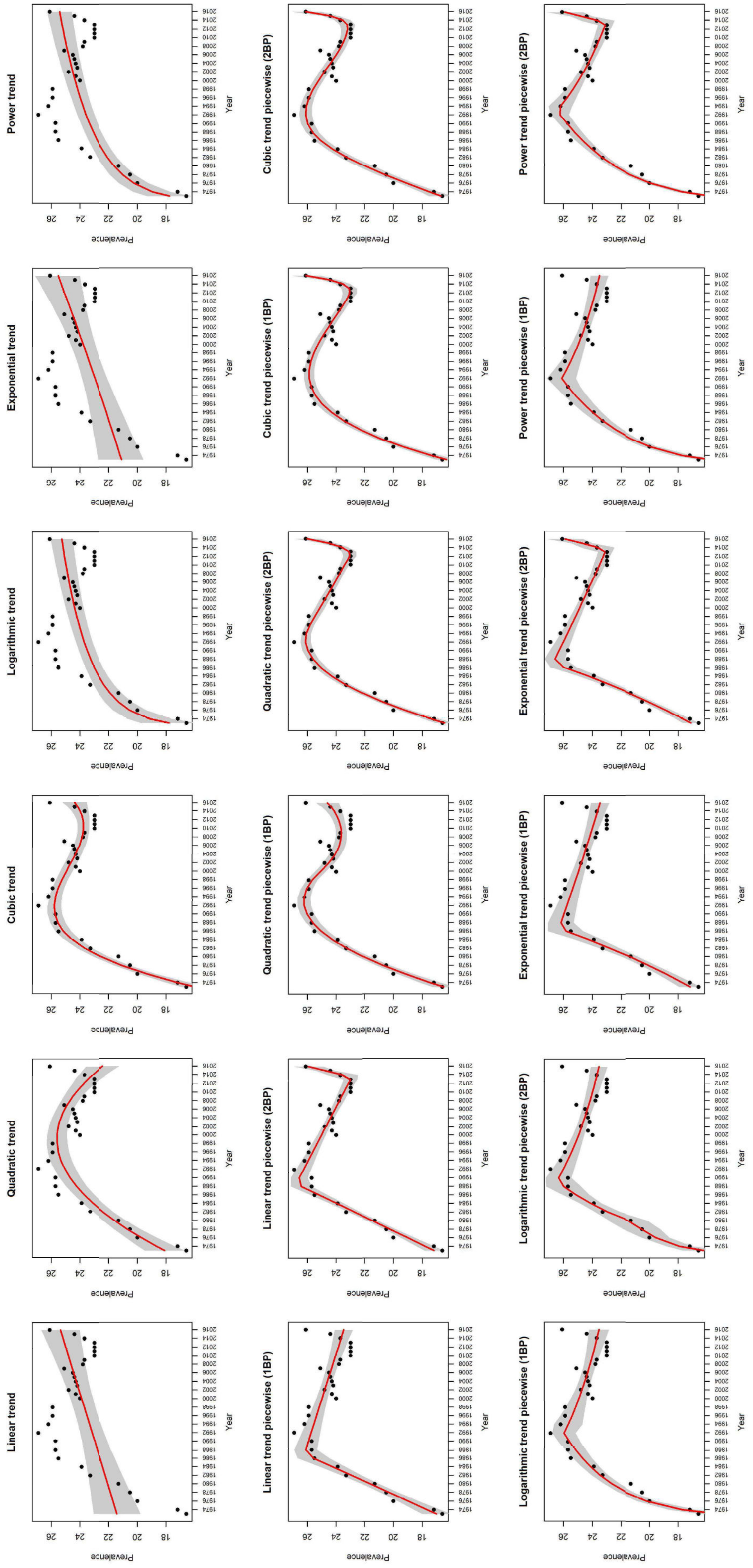

Supplementary Figure 4: Raw and fitted quit ratios from the assessed regression models

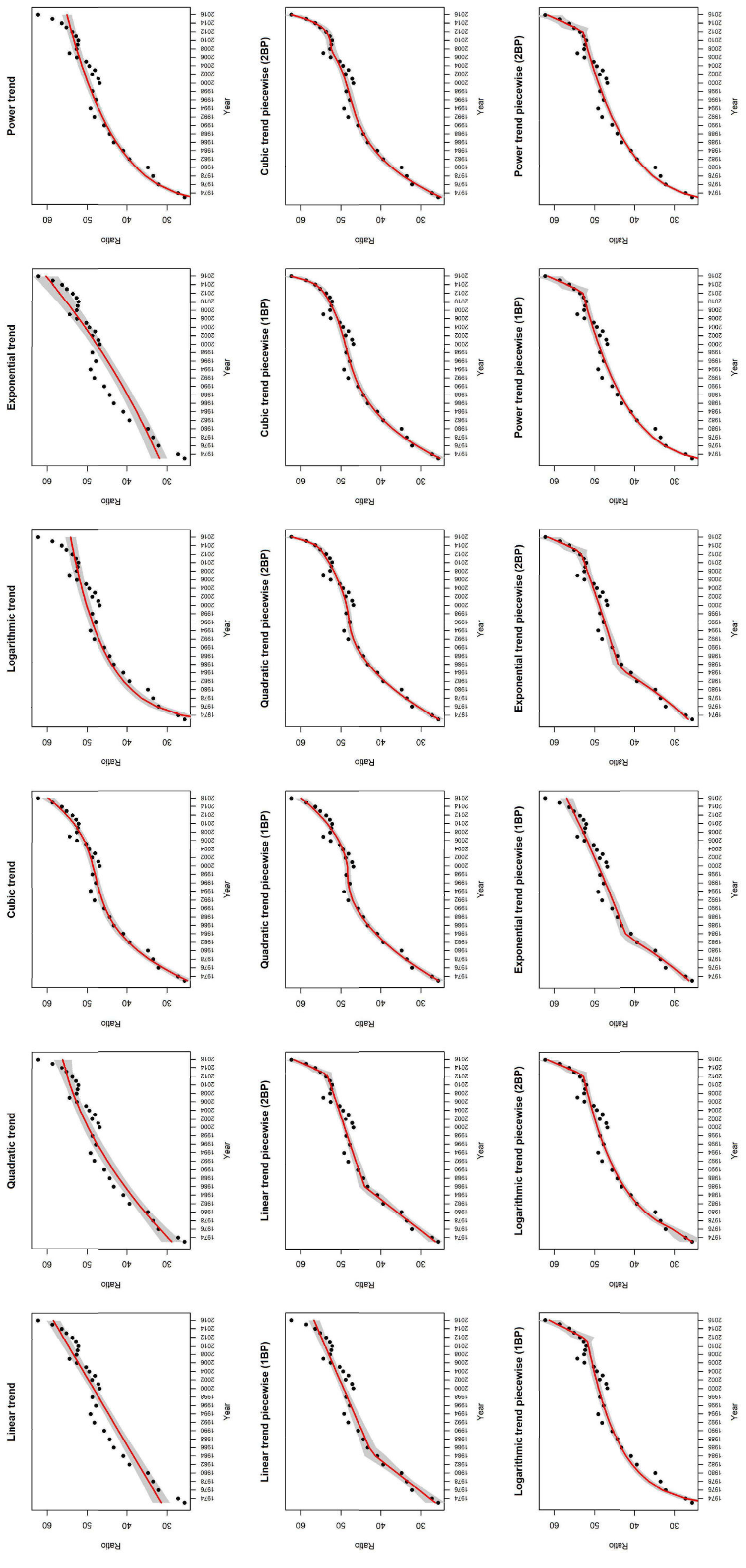

Supplement: Supplementary data [file thoraxjnl-2018-212740supp001.pdf]
